# Supplementary figures and images for: Gut site and sex-specific enrichment of bacterial taxa and predicted metabolic pathways in wild American black bear (Ursus americanus)
Source: PLoS One. 2026 Apr 20;21(4):e0345317. doi: 10.1371/journal.pone.0345317 (PMC13095127; doi:10.1371/journal.pone.0345317)

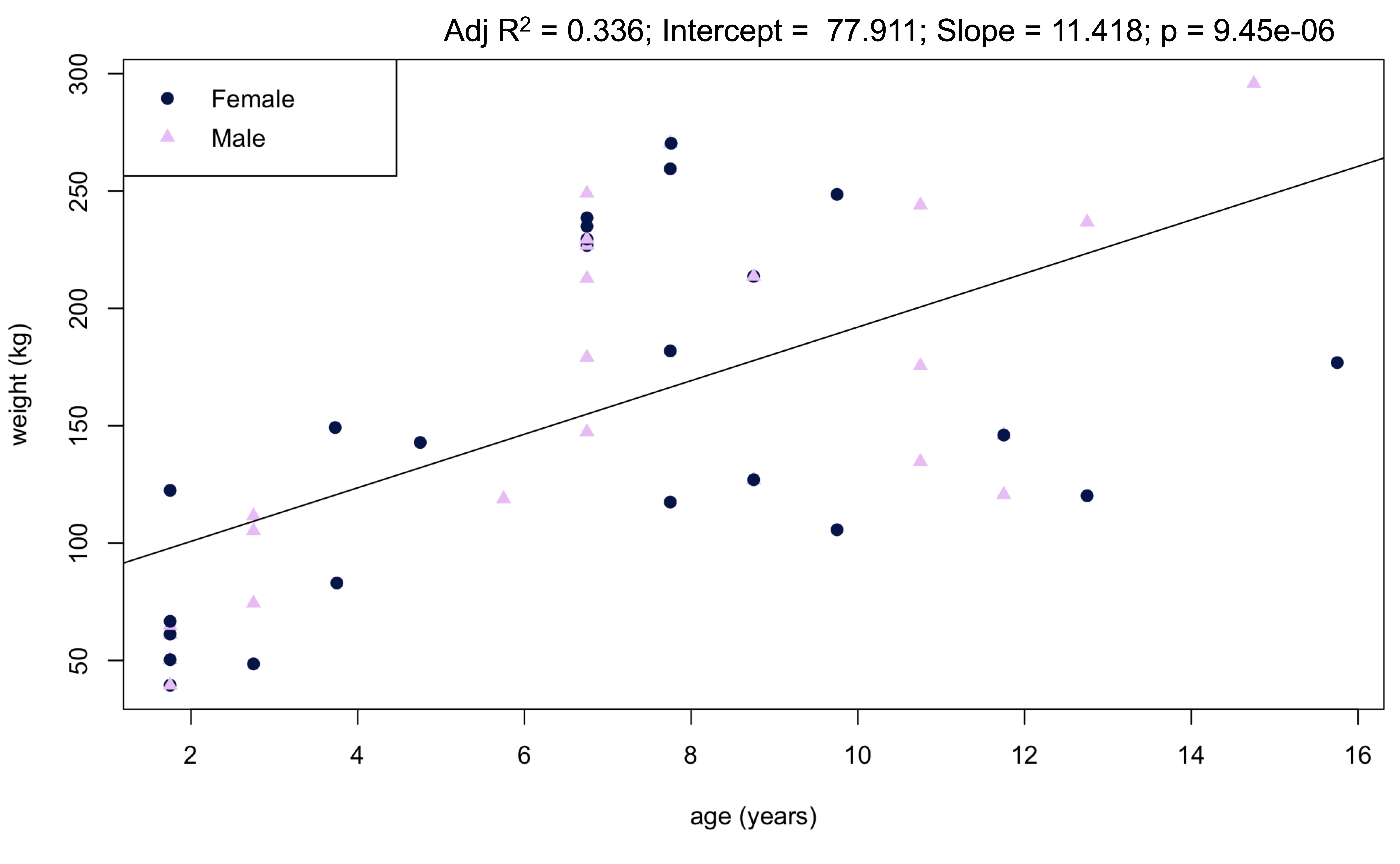

Supplement: S1 Fig — Age was estimated by counting cementum layers from a canine tooth as described by Marks and Erickson [43]. (TIFF) [file pone.0345317.s002.tiff]

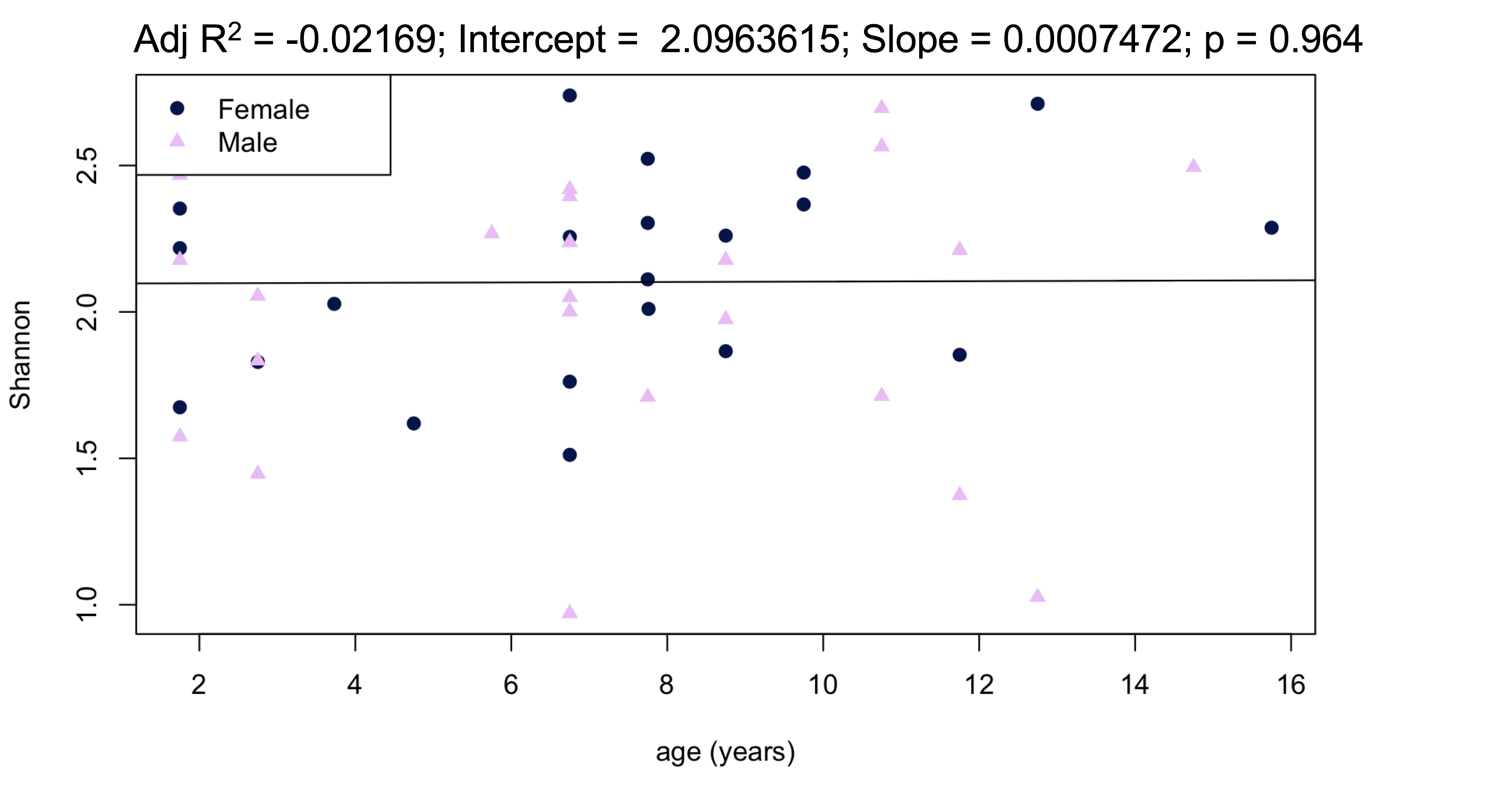

Supplement: S2 Fig — Age was estimated by counting cementum layers from a canine tooth as described by Marks and Erickson [43]. (TIFF) [file pone.0345317.s003.tiff]

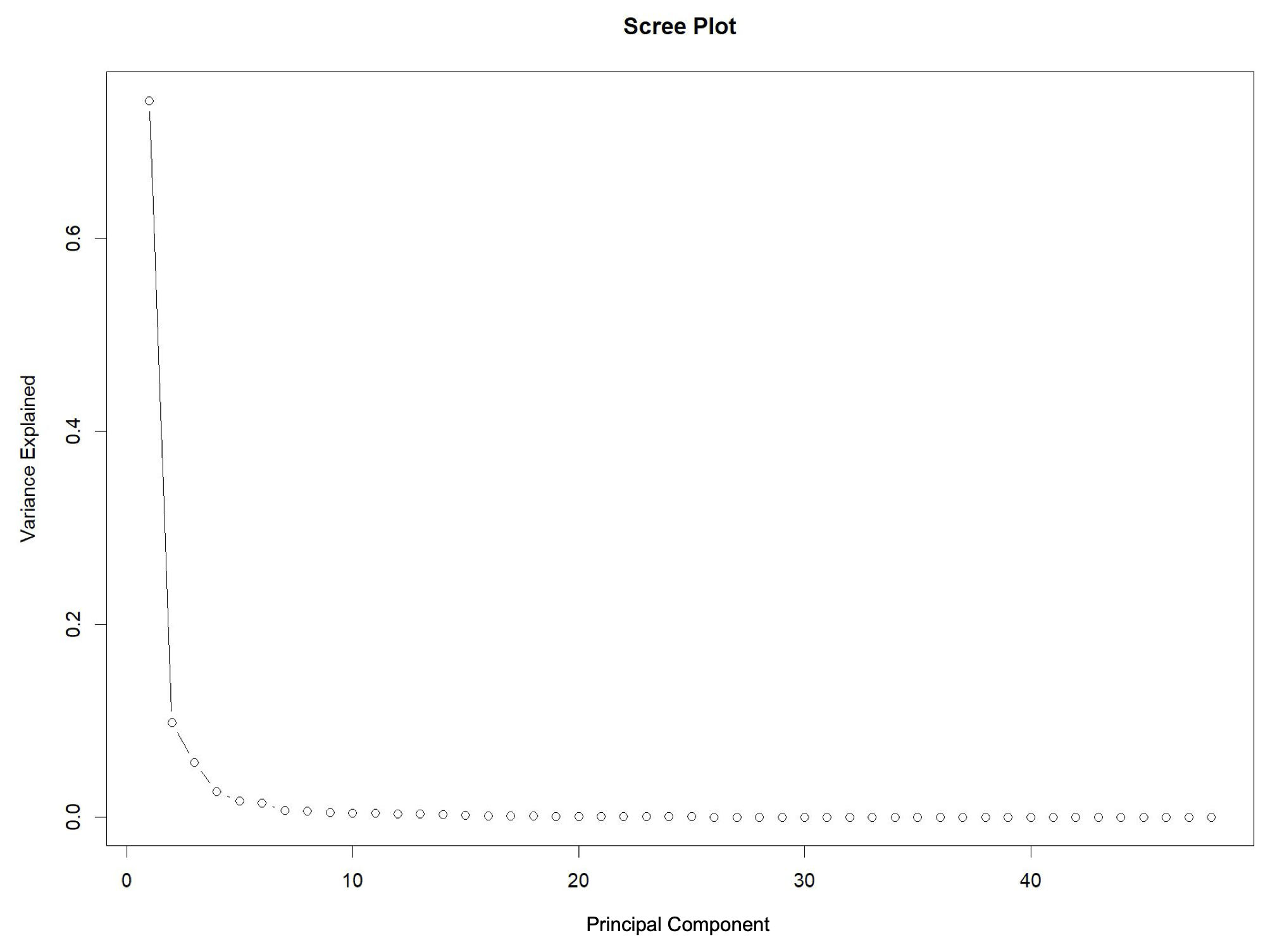

Supplement: S3 Fig — Metabolic pathways were predicted from 16S amplicon sequences using Phylogenetic Investigation of Communities by Reconstruction of Unobserved States (PICRUSt2). (TIFF) [file pone.0345317.s004.tiff]
