# Supplementary material for: Gut site and sex-specific enrichment of bacterial taxa and predicted metabolic pathways in wild American black bear (Ursus americanus)
Source: PLoS One. 2026 Apr 20;21(4):e0345317. doi: 10.1371/journal.pone.0345317 (PMC13095127; doi:10.1371/journal.pone.0345317)
Supplement: Attachment — Submitted filename: Response to reviewers.docx [file pone.0345317.s006.docx]

Dear Dr. Wolf,

On behalf of my co-authors, I am pleased to submit our revised research manuscript titled “Gut site and sex-specific enrichment of bacterial taxa and predicted metabolic pathways in wild American black bear (*Ursus americanus*)” for exclusive publication in *PLOS ONE*. The gut microbiome both complements and shapes host metabolism and health, and provides valuable insights to inform the management of wildlife populations. Black bears’ omnivorous diets, coupled with simple guts and rapid transit times, result in high inter-individual variation that likely supports their generalist niche and adaptive flexibility, and reflects changes in landscape and foraging. Yet we currently lack understanding of how anthropogenic change may impact black bear gut microbiomes in the southeastern United States. To address that gap, students enrolled in an advanced quantitative course on Gut Microbial Ecology at NC State University partnered with researchers at Northern Michigan University and the North Carolina Wildlife Resources Commission. This cross-institutional collaboration not only increases scientific understanding but provides a unique opportunity for students to apply foundational concepts to wildlife management through an authentic course-based research experience. In this study, we examine differences in stable isotope values, gut microbial community composition and predicted metabolic functions among 48 black bears legally harvested in eastern North Carolina.

We are glad that both reviewers “found the manuscript to be of value and interesting”. “It was nicely written, easy to understand and well referenced.” “Overall, this study provides valuable samples on wild American black bears and the results are relevant for the field.” We have addressed all reviewer concerns below (in **bold**), including additional analyses to evaluate differences in community composition associated with age and weight. We hope you will now find our manuscript acceptable for publication in *PLOS ONE*.

We have amended our Funding Statement and Role of Funder Statement as requested:

- Funding was provided by the North Carolina Wildlife Resources Commission and grant funding (W57) through the Federal Aid in Wildlife Restoration Act, popularly known as the Pittman-Robertson Act. In-kind support was provided to DJRL and RH by the College of Arts and Science at Northern Michigan University. There was no additional external funding received for this study. The funders had no role in study design, data collection and analysis, decision to publish, or preparation of the manuscript.

Thank you for considering our work, which includes substantial contributions from undergraduate (E.D.J., D.H., K.H., N.M., H.P., A.M.S., H.T.) and graduate students (T.H., C.I., K.S.), faculty (E.A.M., R.H., D.J.R.L.), and a state regulatory agency (C.O.). Please address all correspondence regarding this manuscript to me at eamckenn@ncsu.edu

Sincerely,

Erin McKenney, MS, PhD (she/they)

Assistant Professor | Director of Undergraduate Programs

Department of Applied Ecology

North Carolina State University

PONE-D-25-36810

Gut site and sex-specific enrichment of bacterial taxa and predicted metabolic pathways in wild American black bear (Ursus americanus)

PLOS ONE

Dear Dr. McKenney,

Thank you for submitting your manuscript to PLOS ONE. After careful consideration, we feel that it has merit but does not fully meet PLOS ONE’s publication criteria as it currently stands. Therefore, we invite you to submit a revised version of the manuscript that addresses the points raised during the review process.

Please submit your revised manuscript by Dec 26 2025 11:59PM. If you will need more time than this to complete your revisions, please reply to this message or contact the journal office at plosone@plos.org. When you're ready to submit your revision, log on to <https://www.editorialmanager.com/pone/> and select the 'Submissions Needing Revision' folder to locate your manuscript file.

Please include the following items when submitting your revised manuscript:

- A rebuttal letter that responds to each point raised by the academic editor and reviewer(s). You should upload this letter as a separate file labeled 'Response to Reviewers'.
- A marked-up copy of your manuscript that highlights changes made to the original version. You should upload this as a separate file labeled 'Revised Manuscript with Track Changes'.
- An unmarked version of your revised paper without tracked changes. You should upload this as a separate file labeled 'Manuscript'.

If you would like to make changes to your financial disclosure, please include your updated statement in your cover letter. Guidelines for resubmitting your figure files are available below the reviewer comments at the end of this letter.

If applicable, we recommend that you deposit your laboratory protocols in [protocols.io](http://protocols.io/) to enhance the reproducibility of your results. Protocols.io assigns your protocol its own identifier (DOI) so that it can be cited independently in the future. For instructions see: [https://journals.plos.org/plosone/s/submission-guidelines#loc-laboratory-protocols](https://track.editorialmanager.com/CL0/https:%2F%2Fjournals.plos.org%2Fplosone%2Fs%2Fsubmission-guidelines%23loc-laboratory-protocols/1/010f019a73269ba4-f22f73f3-4e56-4972-83c0-122e5ea28f29-000000/yW4B4B0OR726wK6FRmbBllRGycKrN3LIVKyFM0dxW-A=236). Additionally, PLOS ONE offers an option for publishing peer-reviewed Lab Protocol articles, which describe protocols hosted on [protocols.io](http://protocols.io/). Read more information on sharing protocols at [https://plos.org/protocols?utm_medium=editorial-email&utm_source=authorletters&utm_campaign=protocols](https://track.editorialmanager.com/CL0/https:%2F%2Fplos.org%2Fprotocols%3Futm_medium=editorial-email%26utm_source=authorletters%26utm_campaign=protocols/1/010f019a73269ba4-f22f73f3-4e56-4972-83c0-122e5ea28f29-000000/10jicEBzYHdZHtYE1Janwdcj1qztpoUpoKyLaaZaDVw=236).

We look forward to receiving your revised manuscript.

Kind regards,

Nathan Wolf
Academic Editor
PLOS ONE

Journal Requirements:

When submitting your revision, we need you to address these additional requirements.

1. Please ensure that your manuscript meets PLOS ONE's style requirements, including those for file naming. The PLOS ONE style templates can be found at

<https://journals.plos.org/plosone/s/file?id=wjVg/PLOSOne_formatting_sample_main_body.pdf> and

[https://journals.plos.org/plosone/s/file?id=ba62/PLOSOne_formatting_sample_title_authors_affiliations.pdf](https://track.editorialmanager.com/CL0/https:%2F%2Fjournals.plos.org%2Fplosone%2Fs%2Ffile%3Fid=ba62%2FPLOSOne_formatting_sample_title_authors_affiliations.pdf/1/010f019a73269ba4-f22f73f3-4e56-4972-83c0-122e5ea28f29-000000/ej3D_r95382Mrz3W3kS47mITqIsKXvpgYD5z4qU9f2g=236).

2. Thank you for stating in your Funding Statement:

“Funding was provided to CO by the North Carolina Wildlife Resources Commission and grant funding (W57) through the Federal Aid in Wildlife Restoration Act, popularly known as the Pittman-Robertson Act. In-kind support was provided to DJRL and RH by the College of Arts and Science at Northern Michigan University.”

Please provide an amended statement that declares *all* the funding or sources of support (whether external or internal to your organization) received during this study, as detailed online in our guide for authors at <http://journals.plos.org/plosone/s/submit-now>. Please also include the statement “There was no additional external funding received for this study.” in your updated Funding Statement.

- **Done**

Please include your amended Funding Statement within your cover letter. We will change the online submission form on your behalf.

- **Done**

3. Thank you for stating the following financial disclosure:

“Funding was provided to CO by the North Carolina Wildlife Resources Commission and grant funding (W57) through the Federal Aid in Wildlife Restoration Act, popularly known as the Pittman-Robertson Act. In-kind support was provided to DJRL and RH by the College of Arts and Science at Northern Michigan University.”

Please state what role the funders took in the study. If the funders had no role, please state: "The funders had no role in study design, data collection and analysis, decision to publish, or preparation of the manuscript."

- **Done**

If this statement is not correct you must amend it as needed.

Please include this amended Role of Funder statement in your cover letter; we will change the online submission form on your behalf.

- **Done**

4. Please be informed that funding information should not appear in the Acknowledgments section or other areas of your manuscript. We will only publish funding information present in the Funding Statement section of the online submission form. Please remove any funding-related text from the manuscript.

- **Done**

5. We note that Figure 9 in your submission contain [map/satellite] images which may be copyrighted. All PLOS content is published under the Creative Commons Attribution License (CC BY 4.0), which means that the manuscript, images, and Supporting Information files will be freely available online, and any third party is permitted to access, download, copy, distribute, and use these materials in any way, even commercially, with proper attribution. For these reasons, we cannot publish previously copyrighted maps or satellite images created using proprietary data, such as Google software (Google Maps, Street View, and Earth). For more information, see our copyright guidelines: <http://journals.plos.org/plosone/s/licenses-and-copyright>.

We require you to either (1) present written permission from the copyright holder to publish these figures specifically under the CC BY 4.0 license, or (2) remove the figures from your submission:

- **We have created a replacement figure in R, and updated the code in our DRYAD submission to include the script used to make the map.**

a. You may seek permission from the original copyright holder of Figure 9 to publish the content specifically under the CC BY 4.0 license.

We recommend that you contact the original copyright holder with the Content Permission Form (<http://journals.plos.org/plosone/s/file?id=7c09/content-permission-form.pdf>) and the following text:

“I request permission for the open-access journal PLOS ONE to publish XXX under the Creative Commons Attribution License (CCAL) CC BY 4.0 (<http://creativecommons.org/licenses/by/4.0/>). Please be aware that this license allows unrestricted use and distribution, even commercially, by third parties. Please reply and provide explicit written permission to publish XXX under a CC BY license and complete the attached form.”

Please upload the completed Content Permission Form or other proof of granted permissions as an "Other" file with your submission.

In the figure caption of the copyrighted figure, please include the following text: “Reprinted from [ref] under a CC BY license, with permission from [name of publisher], original copyright [original copyright year].”

b. If you are unable to obtain permission from the original copyright holder to publish these figures under the CC BY 4.0 license or if the copyright holder’s requirements are incompatible with the CC BY 4.0 license, please either i) remove the figure or ii) supply a replacement figure that complies with the CC BY 4.0 license. Please check copyright information on all replacement figures and update the figure caption with source information. If applicable, please specify in the figure caption text when a figure is similar but not identical to the original image and is therefore for illustrative purposes only.

The following resources for replacing copyrighted map figures may be helpful:

USGS National Map Viewer (public domain): <http://viewer.nationalmap.gov/viewer/>

The Gateway to Astronaut Photography of Earth (public domain): <http://eol.jsc.nasa.gov/sseop/clickmap/>

Maps at the CIA (public domain): <https://www.cia.gov/library/publications/the-world-factbook/index.html> and <https://www.cia.gov/library/publications/cia-maps-publications/index.html>

NASA Earth Observatory (public domain): <http://earthobservatory.nasa.gov/>

Landsat: <http://landsat.visibleearth.nasa.gov/>

USGS EROS (Earth Resources Observatory and Science (EROS) Center) (public domain): <http://eros.usgs.gov/#>

Natural Earth (public domain): <http://www.naturalearthdata.com/>

6. If the reviewer comments include a recommendation to cite specific previously published works, please review and evaluate these publications to determine whether they are relevant and should be cited. There is no requirement to cite these works unless the editor has indicated otherwise.

Additional Editor Comments (if provided):

Thank you for submitting this manuscript to PLOS One. As you will note from the enclosed comments, the Reviewers found the manuscript to be of value and interesting. However, the Reviewers each raised several points that should be considered and potentially revised before the manuscript can be considered for publication.


[Note: HTML markup is below. Please do not edit.]

Reviewers' comments:

Reviewer's Responses to Questions

**Comments to the Author**

1. Is the manuscript technically sound, and do the data support the conclusions?

The manuscript must describe a technically sound piece of scientific research with data that supports the conclusions. Experiments must have been conducted rigorously, with appropriate controls, replication, and sample sizes. The conclusions must be drawn appropriately based on the data presented.

Reviewer #1: Yes

Reviewer #2: Partly

2. Has the statistical analysis been performed appropriately and rigorously?

Reviewer #1: Yes

Reviewer #2: Yes

3. Have the authors made all data underlying the findings in their manuscript fully available?

The [PLOS Data policy](http://track.editorialmanager.com/CL0/http:%2F%2Fwww.plosone.org%2Fstatic%2Fpolicies.action%23sharing/1/010f019a73269ba4-f22f73f3-4e56-4972-83c0-122e5ea28f29-000000/CnKpWdK1YDXpmbgVic41Fzh8xCUnGS0p09rPGe-vZ84=236) requires authors to make all data underlying the findings described in their manuscript fully available without restriction, with rare exception (please refer to the Data Availability Statement in the manuscript PDF file). The data should be provided as part of the manuscript or its supporting information, or deposited to a public repository. For example, in addition to summary statistics, the data points behind means, medians and variance measures should be available. If there are restrictions on publicly sharing data—e.g. participant privacy or use of data from a third party—those must be specified.

Reviewer #1: Yes

Reviewer #2: Yes

4. Is the manuscript presented in an intelligible fashion and written in standard English?

PLOS ONE does not copyedit accepted manuscripts, so the language in submitted articles must be clear, correct, and unambiguous. Any typographical or grammatical errors should be corrected at revision, so please note any specific errors here.

Reviewer #1: Yes

Reviewer #2: Yes

5. Review Comments to the Author

Please use the space provided to explain your answers to the questions above. You may also include additional comments for the author, including concerns about dual publication, research ethics, or publication ethics. (Please upload your review as an attachment if it exceeds 20,000 characters)

Reviewer #1: In this study, McKenney and colleagues analyzed the gut microbiome composition of 48 black bears that were sampled opportunistically at different sites along the gastrointestinal tract. Using a combination of stable isotope analysis and amplicon sequencing, the authors found that bears did not selectively forage on specific types of bait, and microbial diversity did not vary significantly across gut sites. However, they did observe differences in microbial composition and functional potential across gut regions and between male and female bears. Overall, this study provides valuable samples on wild American black bears and the results are relevant for the field.

Comments:

The figures require refinement to improve clarity and readability. In particular:
• All figures: Add axis labels where missing, clearly indicating what each axis represents and including appropriate units of measurement (e.g., "%"). Please also ensure all figures are in high resolution.
• Figure 1: Please increase the size of the corn/peanut icons to make them more easily visible.

**Done.**
• Figure 2: The legend includes colors that are difficult to distinguish. Please use a more distinguishable color palette where each category is clearly separable from adjacent entries. In alternative, reduce the number of categories shown in the legend.

**We have reduced the number of taxa in the legend as suggested.**
• Figures 2 and 3: These figures could be merged into a single figure.

**We appreciate your suggestion. However, Reviewer 2 requested that we re-analyze the gut microbial data based on bear weights and ages. As such, we have re-worked the original figures (as well as the Methods, Results, and Discussion) to incorporate those new analyses and findings. Figures 2 now contains bar charts for age classes as well as gut sites.**
• Figures 4, 5, and 6: These plots could also be combined.

**As above, we appreciate your suggestion; however, Reviewer 2 requested that we re-analyze the gut microbial data based on bear weights and ages. As such, we have re-worked the original figures (as well as the Methods, Results, and Discussion) to incorporate those results. Figure 3 now contains heat maps comparing jejunum vs colon community structure across age classes as well as between males and females. Similarly, Figure 5 contains box plots and Figure 6 contains NMDS plots for age as well as gut site and sex.**
• Figures 7 and 8: Consider combining these into a single figure as they are conceptually related. Also, for Figure 7, please increase the axis font size, as the current labels are too small to be easily read.

**Figure 7 now includes all figures visualizing picrust2 results for predicted metabolic pathways (originally figures 6, 7, and 8). We have also increased the font size for Figure 7 as requested.**
• Figures 6, 7, and 8: Please use the same colors for males/females used in previous figures (the shades of pink and green/blue in these plots are different).

**Done.**

• Figure 9: Add a brief title to each map panel and include a distance scale bar (e.g., how many miles correspond to 1 cm) to help orient the viewer geographically.

**We remade the map in R (now figure 8), which enabled us to color both counties of interest and add a distance scale bar as requested.**

Please provide additional context to the reader on the rationale behind the stable isotopes analysis, and the insights that this kind of analysis provides.

**Done.**

The manuscript would benefit from a deeper discussion of the biological mechanisms that could underlie the observed sex-based differences in microbiome composition and function. Since no sex-specific foraging patterns were detected, the authors should consider and discuss other plausible explanations, such as hormonal differences between males and females that could impact their microbiome.

**We have added a discussion of potential hormonal-mediated physiological cascades that could drive sex-based differences in gut microbiome composition and functionality (page 24).**

The authors should also clarify if and to what extent the month in which the samples were collected could explain the observed variability, as different food types are available throughout the year. How was this possible confounding factor considered in the analyses?

**All sampling was completed in November 2022. By limiting our sampling effort to a single season and year, we avoid any confounding effects associated with longitudinal sampling (i.e., diet * seasonal interactions). We have added this statement to the Methods.**

The authors may also consider adding that the use of metagenomics has the potential to identify antimicrobial resistance genes, a topic of increasingly relevant interest in the context of wildlife sharing habitat with human populations.

**We have added this point to the Discussion.**

Finally, while the authors have shared both the R code and amplicon data via DRYAD, it is recommended that they also deposit the sequencing data on the European Nucleotide Archive (ENA), as standard practice with microbiome data.

**We appreciate the suggestion. We have made the data publicly available on DRYAD, an open data publishing platform and standard practice at NC State University, to ensure that all interested parties can download raw sequences, cleaned ASVs, metadata, and data analysis workflows from a single source.**

Reviewer #2: McKenney et al 2025
I enjoyed the topic and found the research interesting. It was nicely written, easy to understand and well referenced.
The pdf I was given to review did not have line numbers and there were some formatting issues (irregular line spacing, extra spaces after some words, etc.) that I am not sure were due to the journal’s formatting or the article itself.
This is particularly true with the table and figure titles and makes it harder to review and give feedback
However – there are points below that I believe need to be addressed for clarity and increased value to the community.

Abstract –
General comment – the abstract was clear yet vague. The last sentence, for example, how your findings related to conservation and management is not introduced or explained nor easily deduced from the earlier part of the abstract. (If you want this to be about conservation I think the intro and discussion need to discuss this more and make stronger concrete connections). I think your data is solid and trying to link it to the large conservation piece makes it feel more flimsy – thus – stick to what you have more IMO.

**We appreciate this suggestion, and have deleted conservation efforts from the last sentence in the abstract.**

Introduction
- The NC black bear status is from 2010. Is there not more recent information?

**We have confirmed with the NC Wildlife Resources Commission that this is the most recent status report / management plan for NC black bears.**
- Some unnecessary repeating in the introduction (re-read for clarity and to eliminate repeating).

**We have re-read and edited as requested.**
- Page 4 - I would personally not use “we” in scientific report although I know this is becoming more common place. Similarly – I would keep the tense the same throughout (moves from past to present)

**We have edited this paragraph to address inconsistent verb tenses.**
- “these goals” could be better defined

**We have reworded this sentence: “To address these gaps…”.**
- Page 5 – above the table could further explain which isotope values

**We have added a paragraph explaining the stable isotope values on page 4.**
- Page 5 below the table (there is text that I assume is not intended for a find version – says “add new citation from …”

**Thanks for catching this! We have added the citation.**
- Pages 5- 6 – the intro regarding bear baiting could / should be simplified and shortened and focus on the current rules that limit baiting in NC & the repeated use of “licensed hunter” seems odd – perhaps define it once and then move on?

**We have reviewed this section to only use the term “licensed hunter” once, as suggested. We also revised several sentences to be more succinct.**

Also – are these baiting estimates all of NC? I assume differs a bit & even in Terrell Co there are restrictions in which land allows baiting and which doesn’t (thus – where the bear is killed in the Co may also matter)

**The baiting estimates are for all of NC. While the survey results did not compare baiting practice by region or county, our stable isotope figure includes reference values for corn and peanuts (the two most commonly used bait items), enabling us to demonstrate that individual bears in Hyde and Tyrrell counties vary in their dietary consumption and do not tend to cluster near the bait values.**

- Page 6 – when “between” is used to discuss differences between lemur species – should this be “among” thus – more than 2 spots vs just 2?

**Excellent point. We have changed “between” to “among” as suggested.**

Materials and Methods
Why is there yellow highlighting on page 20?

**I highlighted the text to call attention to the reviewer link, and to remind myself to update the statement after the manuscript was accepted. I have removed the highlighting.**

Page 22– text varies from saying “the students did …” vs “we did” – please be consistent

**We have changed “students” to “we” for consistency.**
Page 23 – your figure 1 didn’t include the meat, etc for these comparisons, right? Why not?

**We have updated the stable isotope figure to only include reference values for corn and peanuts. The reference values for processed bait, terrestrial meat, and vegetation were collected for a study of black bears in Michigan and as such are not relevant to the current project in eastern NC. However, peanuts and corn are the top two baits used by hunters in NC.**
Author Contributions portion – some names are not the same font as others (smaller)

**Thank you! We have adjusted the font size for consistency.**
If you want to discuss this population and potential conservation or alterations due to human encroachment, etc – what else can you tell us about these animals? Approximate bear age (data should be available from the teeth) – location found? Weights? Health estimates? Anything? Where they mainly from one of the 2 counties? None of these were from Beaufort Co? Was it private land or public – do we know anything else about these bear?

- **We have removed mention of conservation, as suggested above.**
- **We have re-analyzed the data to evaluate gut microbial communities based on age, both as a continuous variable (in years) and categorizing bears into age-classes (yearling = 1, subadult = 2–3, adult ≥ 4) as previously described by Gillman et al. (2020). We first used linear regression models to verify that bear weights correlate significantly to age. We focus on age for all subsequent analyses because it is tied to gut microbial succession.**
- **All bears were harvested in Hyde and Tyrrell counties.**
- **Hunters harvest on both private and public lands; that information is not collected by the Fish & Wildlife Conservation Commission.**

Results
Sentence 1 – when you state “mid-level” – what is this compared to? Should there be a reference here?

**We have revised this sentence to specify that “bears exhibit variable isotope values across intermediate trophic positions”, and added two citations (Rioux et al. 2020 and Jepsen & Winemiller 2002).**

On my version of the pdf figures 1 and 2 and 7 were slightly blurry and the species names particularly hard to read – perhaps a pdf issue only?

**We have reformatted and reorganized all figures in response to Reviewer 1’s requests and to incorporate the age analyses. This includes increasing text size and resolution.**

Figure 1. The last sentence seems odd on a figure title. I recommend deleting that.

**We have deleted the last sentence as suggested.**

Figure 2 title is in all bold – figure 1 isn’t. Please edit to what the journal wants and be consistent. Also – I like that you note the Eastern NC part in the titles (you don’t do that in all the figures and I would add it to the ones you don’t do it on).

**We have reviewed all figure and table captions to ensure consistency re: bold type and including “eastern NC” in all titles.**

Page 7. Proteo… statement – these differences are statistically different?

**We have included p-values for all significant results. The taxa described in the Results > Community Composition section are not significantly different. However, we did run SIMPER and LEfSe analyses to identify specific taxa that significantly differed between gut sites and sexes (see Materials and Methods > Male vs female black bears). Those significant taxa are specifically addressed in the Results > Beta diversity and Discussion sections.**

Table 2 title ends with “and their respective” which is not clear and needs to be edited

**We have revised the Table 2 caption for clarity.**

In the pdf for my version – the title for Figure 5 and the text run together and need to be separated - or at least I think that is what happened bc the font is not bold for all of it and the 2nd part doesn’t fit as part of a title. However – the same thing happens for Tables 3 & 4 and thus I am not sure if this is a pdf issue or a title issue. Continuing on this theme of potential inconsistencies – Table 5’s title is a different font size (as are some figures).

**We have reviewed all table captions to ensure consistency re: bold type and font size.**

Table 3 could be edited so that some of the words are not on a separate line (making it landscape or just making the columns larger for some and smaller for others)

**We have changed Table 3 and Table 4 to landscape orientation as suggested.**

Page 13 – tense is not consistent

**We have reviewed the entire Results section to ensure that we use past tense consistently.**

Figure 6 has only 8 jejunum samples – I assume the missing sample is discussed somewhere else later? Perhaps discuss here in the results as all for missing samples?

**We have verified that this was a typo: all 9 jejunum samples were included in the principal component analysis.**

Discussion
Sentence one – I would keep this more generic – we do not know that all these animals had enough access to selectively forage – some may have had peanut access and others may not have (could even be that none of them did).

**We have revised the first sentence as suggested.**

Page 15 – The sentence “similar to previous carnivore studies – ref 16” doesn’t make sense as this is not a carnivore reference – do you mean carnivore taxonomically or dietarily? Be clear with these discussions and that may help here as wel.

**We have revised this sentence to “Similar to brown bears (*Ursos arctos*), and specified “taxonomic carnivores with simple gut morphologies” in the following sentence.**

In the same paragraph dogs and cats were discussed as being similar but I would consider altering the cat part out as they are carnivores and you are discussing omnivores dietarily.

**We have deleted cats as suggested.**

Double check that the references are correct in this section, please. For example – when using the reference 13 (about martens) – should this sentence have other refs as well?

**We have added references for black bear and brown bear, which also lack a cecum.**

Prior to ref 33 make sure you are saying the potential dispersal of antibiotic resistance (this was said in the prior sentence but in the 2nd usage it makes it sound like they are definitely dispersing antibiotic resistant strains, and this is not known or studied here). Future research statement is vague and I would suggest deleting it.

**We have specified “potential dispersal” and deleted the final sentence about future research, as suggested.**

Page 16 – you not that body weight gain could be more positive for wildlife – but – it should not be assumed always negative in humans – if that is what you mean in the prior sentence then I would alter that sentence and add a reference.

**We have revised the sentence and added a reference as follows: “However, while obesity increases the risk of other chronic diseases in humans** [**(44)**](https://www.zotero.org/google-docs/?hZ4pYi)**, microbially-facilitated weight gain may have more positive implications for wildlife.”**

Page 17 – the ref 11 as “our expectations” seems odd as written – it seemed like you meant current expectations not ones from a paper 5 years before –

**We have replaced “our expectations” with “previous findings”.**

Discussing age as a limiting factor would be good as well – do you know the ages of any of them? I assume you could get that info from the bear teeth that were collected? I haven’t read the M&M section but also discussing if these were all collected during the same hunting season (as there are two) and in the same year could be potential limitations, etc.

- **We specify in the Materials and Methods that all samples were collected during a single hunting season (November 2022).**
- **We have also re-analyzed the data to evaluate gut microbial communities based on age, both as a continuous variable (in years) and categorizing bears into age-classes (yearling = 1, subadult = 2–3, adult ≥ 4) as previously described by Gillman et al. (2020).**

  References
  Refs are not formatted consistently. Some odd spacing (again – maybe pdf issues – but if not – then fix the punctuation throughout).
  Many do not have pages listed. Some have the paper titles having each word with a capital and some just the 1st word. Some abbreviations are not the same throughout.
  Species names should be italicized. Does the journal not want doi’s listed?
  Does this journal allow personal communication? How do 23 and 27 differ?
  Authors for #42
- **We have ensured that the Word document contains page numbers and line numbers, and checked all abbreviations and species names as requested.**
- **We have also checked all reference formats.**
